# Population Bottlenecks Strongly Affect the Evolutionary Dynamics of Antibiotic Persistence

**DOI:** 10.1093/molbev/msab107

**Published:** 2021-04-19

**Authors:** Etthel M Windels, Richard Fox, Krishna Yerramsetty, Katherine Krouse, Tom Wenseleers, Janne Swinnen, Paul Matthay, Laure Verstraete, Dorien Wilmaerts, Bram Van den Bergh, Jan Michiels

**Affiliations:** 1 VIB Center for Microbiology, Flanders Institute for Biotechnology, Leuven, Belgium; 2 Centre of Microbial and Plant Genetics, KU Leuven, Leuven, Belgium; 3 Inscripta, Boulder, CO, USA; 4 Laboratory of Socioecology and Social Evolution, KU Leuven, Leuven, Belgium

**Keywords:** antibiotic persistence, evolution, population bottlenecks

## Abstract

Bacterial persistence is a potential cause of antibiotic therapy failure. Antibiotic-tolerant persisters originate from phenotypic differentiation within a susceptible population, occurring with a frequency that can be altered by mutations. Recent studies have proven that persistence is a highly evolvable trait and, consequently, an important evolutionary strategy of bacterial populations to adapt to high-dose antibiotic therapy. Yet, the factors that govern the evolutionary dynamics of persistence are currently poorly understood. Theoretical studies predict far-reaching effects of bottlenecking on the evolutionary adaption of bacterial populations, but these effects have never been investigated in the context of persistence. Bottlenecking events are frequently encountered by infecting pathogens during host-to-host transmission and antibiotic treatment. In this study, we used a combination of experimental evolution and barcoded knockout libraries to examine how population bottlenecking affects the evolutionary dynamics of persistence. In accordance with existing hypotheses, small bottlenecks were found to restrict the adaptive potential of populations and result in more heterogeneous evolutionary outcomes. Evolutionary trajectories followed in small-bottlenecking regimes additionally suggest that the fitness landscape associated with persistence has a rugged topography, with distinct trajectories toward increased persistence that are accessible to evolving populations. Furthermore, sequencing data of evolved populations and knockout libraries after selection reveal various genes that are potentially involved in persistence, including previously known as well as novel targets. Together, our results do not only provide experimental evidence for evolutionary theories, but also contribute to a better understanding of the environmental and genetic factors that guide bacterial adaptation to antibiotic treatment.

## Introduction

An antibiotic treatment that is lethal for the majority of a bacterial population often leaves a small number of bacteria unaffected. These persister cells result from a phenotypic switch to an antibiotic-tolerant state and can re-establish a population after treatment, potentially causing antibiotic therapies to fail and infections to relapse (Fisher et al. 2017; Dewachter et al. 2019). Moreover, it has recently been hypothesized that the clinical burden of persistence reaches beyond recurrent infections, as persistence might also accelerate the emergence of resistance (Levin-Reisman et al. 2017; Sebastian et al. 2017; Bakkeren et al. 2019; Windels, Michiels, Fauvart, et al. 2019; Windels, Michiels, Van den Bergh, et al. 2019).

Alarmingly, a growing body of evidence shows that persistence can very rapidly evolve to high frequency in a population. Repeated exposure to antibiotics results in bacterial strains that accommodate up to a 1,000 times more persisters than their progenitors (Fridman et al. 2014; Mechler et al. 2015; Michiels et al. 2016; Van den Bergh et al. 2016; Khare and Tavazoie 2020; Sulaiman and Lam 2020). The antibiotic treatment frequency is known to affect the rate of persistence evolution (Van den Bergh et al. 2016), but the role of other parameters remains poorly understood. Here, we investigate how population bottlenecks alter the evolutionary dynamics of persistence. Bottlenecks are highly prevalent during infection by many pathogens, especially during host-to-host transmissions and antibiotic treatment (LeClair and Wahl 2018). Host colonization can be initiated by a single genetic variant of a pathogen (Moxon and Murphy 1978; Rubin 1987; Abrahams et al. 2009), whereas other pathogens need to attack their host en masse in order to have a few individuals surviving the host defense mechanisms (Ribet and Cossart 2015). In both cases, the population is forced through a small bottleneck that considerably affects subsequent within-host infection dynamics and evolution. Multihost pathogens must additionally overcome small bottlenecks while colonizing and adapting to a new host (Bacigalupe et al. 2019). After colonization, pathogens frequently encounter similar bottlenecking events. For example, host–parasite interactions are generally characterized by significant fluctuations in population size (Gokhale et al. 2013), and the intracellular uptake of bacteria or an attack by antibiotics or the immune system may strongly reduce the size of bacterial populations (Abel et al. 2015).

Bottlenecks are known to influence the evolutionary dynamics mainly by increasing the impact of genetic drift and reducing the mutational supply rate. How the overall fitness of evolved strains is affected by frequent bottlenecking events is expected to depend on the shape of the underlying fitness landscape, which can be single-peaked (i.e., smooth) or characterized by multiple peaks (i.e., rugged). Large bottlenecks, which tend to preserve more genetic diversity, are assumed to provoke faster adaptation when the fitness landscape is smooth. In contrast, evolution on rugged landscapes relies on specific epistatic interactions which might be easier to access when small bottlenecks are applied (Rozen et al. 2008; Handel and Rozen 2009). However, small bottlenecks result in a more stochastic scanning of the mutational space, with the potential side effect of populations ending up at local, suboptimal fitness peaks (Weinreich and Chao 2005).

The fitness landscape describing genotype–phenotype relations for persistence is not well defined, complicating the prediction of bottlenecking effects on the evolutionary dynamics of persistence. By exposing populations to a range of bottleneck sizes, we aimed to uncover these effects as well as to indirectly explore the topography of the persistence fitness landscape. To this end, we first established a high-throughput protocol to evolve many parallel *Escherichia coli* populations under intermittent antibiotic treatment, with varying bottleneck sizes. Next, we adopted a novel, highly multiplexed, trackable genome editing technique and subjected genome-wide knockout (KO) libraries to a range of bottlenecks. The extensive set of resulting data indicates that bottlenecking events considerably affect the evolutionary dynamics of antibiotic persistence. Populations that are forced through small bottlenecks adapt more slowly to daily antibiotic exposure and tend to display lower and more diverse persister levels. Accordingly, we found that small bottlenecks reduce within-population genetic diversity and promote population divergence, with parallel populations ending up at different locations of increased persistence on the fitness landscape. The heterogeneous evolutionary outcomes found in these populations suggest that the persistence fitness landscape is rugged and reveal a novel set of genotypes underlying increased persistence.

## Results

### Bottlenecking Restrains the Evolution of Persistence

**Fig. 1.**
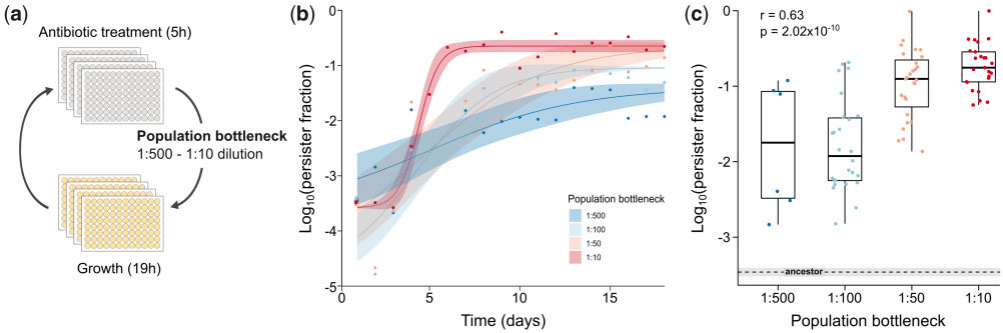
Bottlenecking restrains the evolution of persistence. (*a*) Schematic workflow of the evolution experiments performed in this study. Stationary phase populations of *E. coli* were exposed to daily, high-dose amikacin treatment (5 h), intermitted with periods of growth (19 h) in order to select for persistence. Population bottlenecks were enforced by diluting cultures after treatment, with dilution factors ranging from 1:500 to 1:10. (*b*) Average time course of persister fractions of evolving populations, with a sigmoidal fit and 95% CI (shading) describing the evolutionary trajectories (*n* = 7–27; see Materials and Methods). The rate of adaptation positively correlates with the bottleneck size (supplementary fig. S5, Supplementary Material online). (*c*) Persister fractions of individual end populations positively correlate with the bottleneck size (Spearman rank correlation: *r* = 0.63; *P* = 2.02 × 10^−10^; *n* = 7–27), whereas the variance among parallel populations decreases with increasing bottleneck size (Spearman rank correlation: *r* = −1.00; *P* = 0.08) (dashed line: mean of ancestor; gray shading: 95% CI of ancestor).

Similar to previous experiments by Van den Bergh *et al.* (2016), we selected for persistence by exposing stationary phase populations of *E. coli* to daily, high-dose amikacin treatments, intermitted with periods of growth (fig. 1*a*; Van den Bergh et al. 2016). However, we drastically increased the throughput of this protocol by scaling down culture volumes (see Materials and Methods). We anticipated that reducing the population size would not only improve our capacity to evolve many populations in parallel, but also intensify the potential effects of bottlenecking events. Population bottlenecks were enforced by high-dose antibiotic treatments as well as through the process of serial transfer when starting new cycles of evolution, which involved dilution of antibiotic-treated populations in fresh medium (Van den Bergh et al. 2018). In order to investigate the effect of population bottlenecks on the rate and extent of persistence evolution, the dilution was varied from 1:500 to 1:10. In the first cycle of the experiment, this range approximately corresponded to 60 (1:500) up to 3,000 (1:10) viable cells that were transferred to the next cycle, with these numbers increasing as antibiotic survival improved. Forty parallel populations were propagated per condition. The majority of strongly diluted populations went to extinction (supplementary fig. S1, Supplementary Material online), presumably as a joint result of strong antibiotic selection and extremely small bottlenecks, whereas we observed around 25% extinction in the other conditions. In populations that survived the 18 days of evolution, the fraction of persister cells showed a 10- to 1,000-fold increase over the course of the experiment, which is in accordance with previous studies (Michiels et al. 2016; Van den Bergh et al. 2016; Khare and Tavazoie 2020; Sulaiman and Lam 2020) (fig. 1*b* and *c* and supplementary fig. S2, Supplementary Material online). The minimum inhibitory concentration (MIC) remained unaffected (supplementary fig. S3, Supplementary Material online), whereas the time-kill curves of evolved populations are biphasic (supplementary fig. S4, Supplementary Material online). Together, these observations point toward increased persistence without an increase in resistance. Notably, both the rate of persistence evolution and the final persister fraction are correlated with the bottleneck size, with smaller bottlenecks resulting in slower evolution and a more limited increase of the persister fraction in evolved populations (fig. 1*b* and *c* and supplementary fig. S5, Supplementary Material online; Spearman rank correlation: *r *= 0.63; *P* = 2.02 × 10^−10^). Furthermore, between-population heterogeneity is stronger among parallel populations evolved with a smaller bottleneck, as demonstrated by the larger variation in persister fractions of end populations (fig. 1*c*; Spearman rank correlation: *r* = −1.00; *P* = 0.08). This heterogeneity hints at the presence of multiple peaks on the persistence fitness landscape, and might thus reflect a rugged topography.

### Bottlenecking Negatively Affects the Fitness of Evolved Populations

**Fig. 2.**
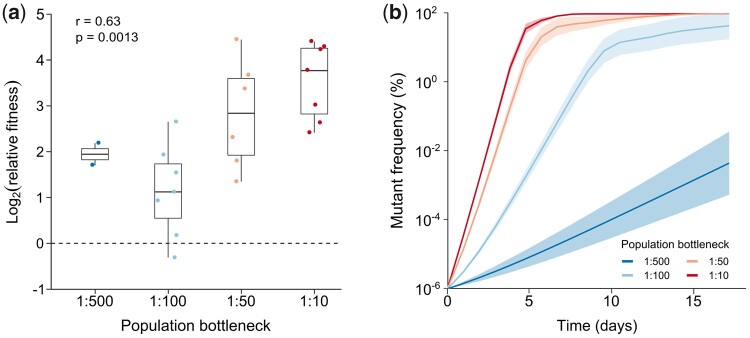
Bottlenecking negatively affects the relative fitness of evolved populations. (*a*) Relative fitness of evolved populations determined by head-to-head competition against the ancestral strain. Evolved populations show a strong fitness advantage over the ancestral strain (two-sided *t*-test: *P* = 0.00013). Furthermore, the relative fitness is positively correlated with the bottleneck size (Spearman rank correlation: *r* = 0.63; *P* = 0.0013) (dashed line: ancestral reference). (*b*) Simulated evolutionary trajectories representing the spread of a single hypothetical mutant in an ancestral population exposed to daily bottlenecks (see Materials and Methods). Evolutionary trajectories were simulated for each population for which the relative fitness was measured, and the average trajectories (±SEM) per bottleneck are shown. The lower the relative fitness and the smaller the bottleneck, the slower the frequency of the mutant in the population increases.

The relative fitness of evolved populations was determined by competing them against the ancestral strain during one round of selection. To this end, evolved populations were mixed with the ancestral strain at a 50:50 ratio and grown overnight, treated with antibiotics for 5 h and again grown overnight. The relative frequencies of both strains were evaluated after the first and second growth phase by fluorescence microscopy (fig. 2*a*). The relative fitness reflects the combined effects of our selection regime on antibiotic survival and growth. As expected, evolved populations exhibit a significant fitness advantage over the ancestral strain under selective conditions (*P* = 0.00013). Furthermore, our data show a positive correlation between the bottleneck size and the relative fitness of evolved strains (Spearman rank correlation: *r* = 0.63; *P* = 0.0013). In order to visualize the effect of the relative fitness on the evolutionary trajectories, we simulated the spread of a single mutant in an ancestral population under conditions applied during experimental evolution, with the relative fitness of the mutant given by each of the experimentally measured values (fig. 2*b*; see Materials and Methods). The simulated trajectories per bottleneck demonstrate that the mutant frequency increases more rapidly when the relative fitness is higher and the population bottleneck is larger.

### Growth Characteristics of Evolved Populations Are Correlated with the Bottleneck Size

**Fig. 3.**
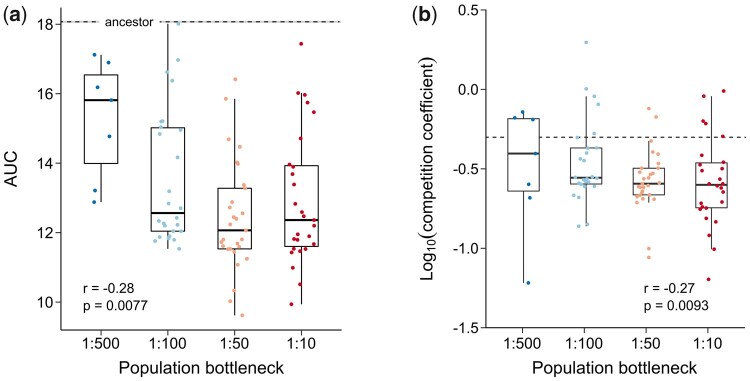
Growth characteristics of evolved populations are correlated with the bottleneck size. (*a*) AUC calculated from growth curves of evolved populations grown in monocultures. The AUC of evolved populations is significantly lower than the AUC of the ancestral strain (dashed line) (two-sided *t*-test: *P* < 10^−12^). Furthermore, the AUC of monocultures negatively correlates with the bottleneck size (Spearman rank correlation: *r* = −0.28; *P* = 0.0077) (dashed line: mean of ancestor; gray shading: 95% CI of ancestor). (*b*) Competition coefficients of evolved populations under growth-favorable conditions, calculated from the AUC of monocultures and the AUC of mixed cultures. The competition coefficient is negatively correlated with the bottleneck size (Spearman rank correlation: *r* = −0.27; *P* = 0.0093) (dashed line: competition coefficient = 0.5).

One cycle in our evolution experiments corresponds to a 5-h antibiotic treatment of stationary phase populations, followed by dilution in fresh medium and growth overnight. This regime might not merely select for increased antibiotic survival, but also affect the growth characteristics of evolving populations (Van den Bergh et al. 2016). As growth could be an important component determining the fitness of end populations, we could expect a positive correlation between the bottleneck size and the growth rate. On the other hand, previous studies have suggested that high persistence is associated with a fitness cost reflected in a growth deficit (Stepanyan et al. 2015; Van den Bergh et al. 2016). To evaluate growth of our evolved populations in fresh, antibiotic-free medium, we followed optical densities of all populations over time. The resulting growth curves did not always follow a characteristic pattern that could be described by relevant growth parameters, presumably as a result of nutrient shifts occurring in complex growth media (supplementary fig. S6, Supplementary Material online). Instead, we used the area under the curve (AUC) as a quantitative parameter to summarize overall growth. Notably, the AUCs of all evolved populations in monoculture are considerably diminished as compared with the ancestral strain (*P* < 10^−12^; fig. 3*a*). This is mainly due to a reduced growth rate after the shift (supplementary fig. S6, Supplementary Material online), and points toward a trade-off between the ability of a population to survive an antibiotic treatment and its growth rate under favorable conditions, as has been suggested before (Stepanyan et al. 2015; Van den Bergh et al. 2016). Moreover, the AUC negatively correlates with the bottleneck size (Spearman rank correlation: *r* = −0.28; *P* = 0.0077), implying that the adverse effect of the selection regime on growth is positively correlated with its beneficial effect on antibiotic survival.

Recently, a method has been proposed to derive competition parameters from growth in mixed cultures (Ram et al. 2019). Inspired by this work, we measured growth of evolved populations mixed in equal ratios with the ancestral strain. The AUCs of these mixed cultures follow a pattern that is similar to the monocultures (supplementary fig. S7, Supplementary Material online), although lacking statistical significance due to the equalizing effect of the ancestral strain (Spearman rank correlation: *r* = −0.099; *P* = 0.35). As a growth or competition model could not be fitted to our data, we used the AUC of monocultures and mixed cultures to derive a competition coefficient for each population (fig. 3*b*) (Ram et al. 2019). This coefficient is a measure for the competitive (dis)advantage of evolved populations over the ancestral strain under growth-promoting conditions and is also negatively correlated with the bottleneck size (Spearman rank correlation: *r* = −0.27; *P* = 0.0093). As the bottleneck size is positively correlated with antibiotic survival on the one hand, and negatively correlated with growth on the other hand, we can conclude that the overall fitness of evolved populations is mainly determined by their ability to survive a high-dose antibiotic treatment. Additionally, these data suggest that the growth cost under antibiotic-free conditions increases with the extent of adaptation to frequent antibiotic exposures. In agreement with previous studies, this cost implies a pleiotropic effect of persistence mutations (Stepanyan et al. 2015; Van den Bergh et al. 2016).

### Bottlenecking Affects the Genetic Composition of Populations and Promotes Between-Population Heterogeneity

**Fig. 4.**
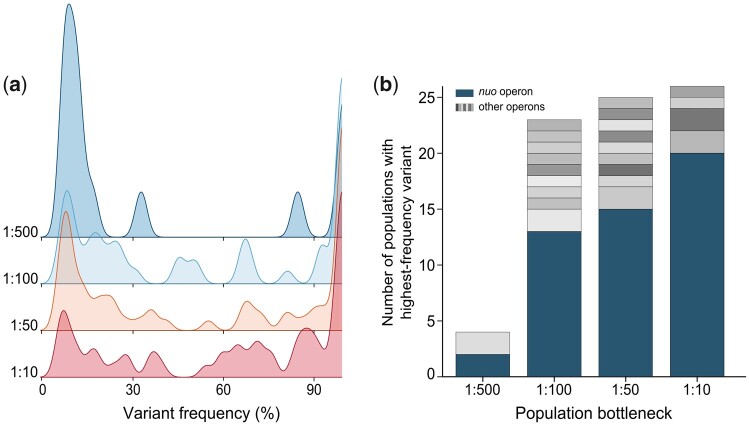
Bottlenecking affects the genetic composition of evolved populations and promotes between-population heterogeneity. (*a*) Distribution of variant frequencies per population. The peak height represents the number of variants with a certain frequency within a population, normalized over the total number of populations. Small-bottleneck populations contain many low-frequency variants and a few high-frequency variants, whereas large-bottleneck populations contain much more intermediate-frequency variants due to clonal interference. (*b*) Number of populations in which a certain variant attained the highest frequency. Different colors represent different operons. Parallel populations exposed to large bottlenecks more often contain similar variants, whereas small-bottleneck populations show more divergence (Spearman rank correlation: *r *= 1.00; *P* = 0.083).

Population bottlenecks are known to affect the evolutionary dynamics by increasing the level of genetic drift and reducing the mutational supply rate. In order to study the impact of bottlenecking on the genetic diversity within and between our evolved populations, we first sequenced the genomes of all evolved end populations. Data on the frequency of each mutated gene within populations reveal a considerable effect of bottlenecking on the population composition. Populations exposed to small bottlenecks consist of a few high-frequency mutants and many low-frequency mutants. In contrast, the composition of large-bottleneck populations is more uniformly distributed, with more variants reaching intermediate frequencies, presumably as a result of clonal interference (fig. 4*a*). Many evolved populations contain mutations in the *nuo* operon. This operon consists of 13 genes that encode NADH:ubiquinone oxidoreductase, that is, complex I of the respiratory chain (Efremov and Sazanov 2011), and has previously been identified as a genetic determinant of persistence (Shan et al. 2015; Van den Bergh et al. 2016; Sulaiman and Lam 2020). Notably, our high-throughput protocol allowed us to identify mutations in a much larger number of genes within this operon than previously reported, as well as in other operons that have not yet been associated with persistence (supplementary fig. S11*a* and table S1, Supplementary Material online). More importantly, our data show that high-frequency mutations in parallel populations exposed to large bottlenecks more often occur in the same operon. In 77% of the large-bottleneck populations (1:10), *nuo* mutations reached the highest frequency (fig. 4*b*). Smaller bottlenecks resulted in fewer populations containing these variants, whereas alternative evolutionary outcomes were attained (Spearman rank correlation: *r* = 1.00; *P* = 0.083). These alternative outcomes indicate that we uncovered additional peaks, or slopes leading toward additional peaks, on the persistence fitness landscape. Furthermore, when combining our phenotypic and genetic data, we can conclude that the genetic drift and low genetic diversity associated with small bottlenecks tend to inflict swift fixation of mutations with potentially suboptimal fitness effects, and consequently lead to stronger population divergence. In contrast, the higher mutational supply rate associated with larger bottlenecks results in a higher degree of clonal interference, as is reflected in the amount of intermediate-frequency mutants. These populations more often attain similar evolutionary outcomes which are presumably located around the nearest fitness optimum.

**Fig. 5.**
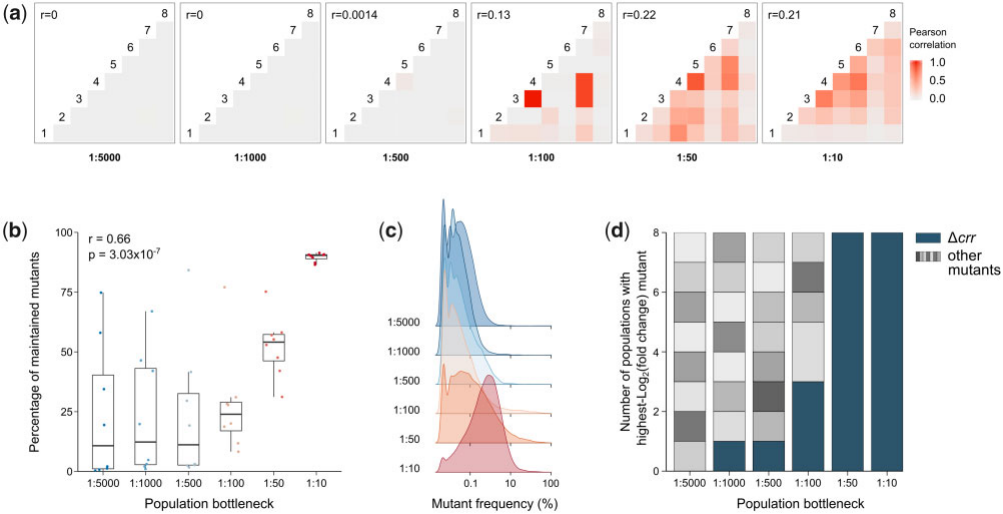
Bottlenecking affects the composition of genome-wide gene KO libraries after antibiotic selection and promotes between-population heterogeneity. (*a*) Correlation plots of the genetic composition of eight parallel populations, obtained by subjecting gene KO libraries to two rounds of antibiotic selection. The composition of the populations after selection was determined by calculating the frequency of each mutant based on raw sequencing read counts. Small population bottlenecks result in weaker correlations between parallel populations (*r* = Pearson correlation averaged over all comparisons). No negative correlations were observed. (*b*) The genetic diversity, represented as the percentage of mutants maintained in a population after selection, is positively correlated with the bottleneck size (Spearman rank correlation: *r* = 0.66; *P* = 3.03 × 10^−7^). (*c*) Distributions of mutant frequencies within populations. The peak height represents the number of mutants with a certain frequency within a population, normalized over the total number of populations. Small-bottleneck populations mainly contain low-frequency mutants, whereas the distribution shifts to higher frequencies as the bottleneck increases. (*d*) Number of populations in which a certain mutant shows the highest absolute log_2_(fold change) after selection, based on raw read counts. Different colors represent different mutants. Parallel populations exposed to large bottlenecks often contain similar mutants, whereas small-bottleneck populations show a larger between-population heterogeneity (Spearman rank correlation: *r* = 0.97; *P* = 0.0012).

In the above-described evolution experiments, we started from an isogenic population and let genetic diversity emerge as a result of *de novo* mutations. In contrast to this approach, the next set of experiments was initiated from a comprehensive pool of genetically diverse mutants, which was then narrowed down by selection. To this end, we adopted the novel, massively parallel, CRISPR-based genome engineering Onyx technology. Two different Onyx libraries were constructed (see Materials and Methods). The first library contains 4,128 different *E. coli* gene KO mutants and the second library includes 87 noncoding RNA (ncRNA) KO mutants. All mutants are trackable by barcodes that are uniquely associated with each mutation. The pooled libraries were subjected to two rounds of our selection regime with a range of bottleneck sizes, and the frequency of each mutant was tracked over time. Population bottlenecks were varied from 1:5,000 to 1:10 dilution during serial transfer. For each bottleneck, eight parallel populations were initiated from each of the two libraries. In contrast to the gene KO library, populations initiated from the ncRNA KO library rapidly went extinct when extreme bottlenecks of 1:5,000 and 1:1,000 were applied. This might be the result of the lower library complexity, implying a potentially lower number of beneficial mutations. Yet, it might also suggest that knocking out ncRNAs generally has a more detrimental effect on survival than knocking out genes. We determined the effect of bottlenecking on the between-population heterogeneity by calculating the correlation between the composition of parallel populations. Notably, we found that small bottlenecks significantly weaken the correlation between parallel populations, and thus promote divergence of populations under selection (fig. 5*a* and supplementary figs. S8, S9, and S10*a*, Supplementary Material online). In contrast to this effect on the interpopulation genetic diversity, the intrapopulation diversity is reduced by bottlenecking. Indeed, the percentage of mutants that were maintained after selection decreases with decreasing bottleneck size, although a plateau seems to be reached for extremely small bottlenecks (fig. 5*b* and supplementary fig. S10*b*, Supplementary Material online; Spearman rank correlation: *r* = 0.66; *P* = 3.03 × 10^−7^). Furthermore, the within-population diversity, which can be described by the distribution of mutant frequencies after selection, is considerably impacted by the bottleneck size (fig. 5*c* and supplementary fig. S10*c*, Supplementary Material online). Small bottlenecks result in a skewed genotype distribution, with many low-frequency mutants and very few mutants with a high frequency. As the bottleneck increases, the distribution becomes more uniform and shifts to higher frequencies. In accordance with the sequencing data from evolution experiments, these barcode sequencing data indicate that small bottlenecks result in reduced within-population genetic diversity and a more random selection of mutations, causing populations to end up at more diverse locations on the adaptive landscape.

By comparing DESeq2 normalized read counts before and after selection, we identified mutants that were significantly enriched or depleted in populations after selection. Although the randomness introduced by bottlenecking diversified parallel populations selected with the same bottleneck, we considered these populations as biological replicates to be able to discover statistically significant trends. For the low-complexity ncRNA KO library, this resulted in a list of significantly depleted mutants per bottleneck (supplementary fig. S11*b* and table S2, Supplementary Material online). The lack of statistically significant enrichments in these populations further supports the aforementioned hypothesis of ncRNAs having generally deleterious effects on survival when knocked out. Performing the analysis over all bottlenecks introduced more variation between replicate populations and therefore led to fewer significant results. Only Δ*micC* and Δ*ohsC* were found to be significantly depleted over all populations. MicC is a small RNA that controls the expression of the outer membrane pore OmpC, and OhsC negatively regulates the expression of the toxic peptide ShoB (Chen et al. 2004; Fozo et al. 2008). For the gene KO library, comparing the DESeq2 normalized read counts resulted in fewer significant hits (supplementary fig. S11*b* and table S3, Supplementary Material online). This can be attributed to the stronger effect of bottlenecking on the between-population heterogeneity, resulting from the higher complexity of the library. Moreover, the detection of depletions is considerably complicated by the bottlenecking events, which cause a major part of the library to be lost. When performing the analysis over all bottlenecks, only Δ*crr* shows a strongly significant enrichment. *crr* encodes the enzyme EIIA^Glc^, a phosphotransfer protein involved in the uptake and phosphorylation of various sugars (Boos et al. 1990; Buhr et al. 1994) as well as the regulation of carbon metabolism (Deutscher et al. 2006). The Δ*crr* mutant is strongly enriched in 21 out of 48 populations. Furthermore, this mutant is most frequent in all populations subjected to large bottlenecks (1:50 and 1:10), whereas small-bottleneck populations display much more variability in the outcome of selection (fig. 5*d* and supplementary fig. S10*d*, Supplementary Material online; Spearman rank correlation: *r* = 0.97; *P* = 0.0012). These results again suggest that bottlenecking promotes population divergence, confirming our earlier observations.

## Discussion

In this study, we used high-throughput, miniaturized experimental evolution, CRISPR-based pooled mutants screens, and massive next-generation sequencing to investigate how population bottlenecks affect the evolutionary dynamics of antibiotic persistence. In accordance with theoretical hypotheses, we found that extreme bottlenecking significantly impairs the adaptive potential of bacterial populations, with adaptation occurring at a slower rate and halting at lower persister levels. Furthermore, small bottlenecks increase the between-population heterogeneity while decreasing the genetic diversity within populations. Our results agree with the notion of a persistence fitness landscape that is rugged (i.e., containing a multitude of potential ways to improve survival under the selective conditions) and allowed us to identify novel genes and ncRNAs that are potentially involved in antibiotic persistence.

Bottlenecking is experienced by many bacterial pathogens during infection and has already been identified as an important constraint on the rate of evolutionary adaptation (Wahl and Gerrish 2001; Wahl et al. 2002; Willi et al. 2006; Vogwill et al. 2016; Garoff et al. 2020). Here, we tested existing evolutionary hypotheses regarding population bottlenecks on the specific, clinically relevant case of antibiotic persistence. Moreover, by exposing populations to small bottlenecks, we aimed to introduce stochasticity in the followed evolutionary trajectories, in order to get a grasp of the topography of the fitness landscape associated with persistence. To this end, we developed a protocol for high-throughput experimental evolution of persistence. This protocol is largely based on a previously established method (Van den Bergh et al. 2016), with the important improvement that it allows the processing of hundreds of populations in parallel due to a drastic reduction of the population size. The selection regime consists of a daily, high-dose antibiotic treatment intermitted with growth in antibiotic-free medium, and specifically selects for mutants with elevated persister levels. This setup is highly appropriate to investigate the effects of bottlenecking, as the high-dose antibiotic treatments considerably reduce the population size, whereas the serial transfer events mimic bottlenecks inflicted by host-to-host transmissions.

Fitness in our selection regime is mainly determined by the ability to survive a high-dose antibiotic treatment, corresponding to the persister level of the population. Furthermore, we found that increased persistence is associated with impaired growth, pointing toward a pleiotropic effect of persistence mutations.

**Fig. 6.**
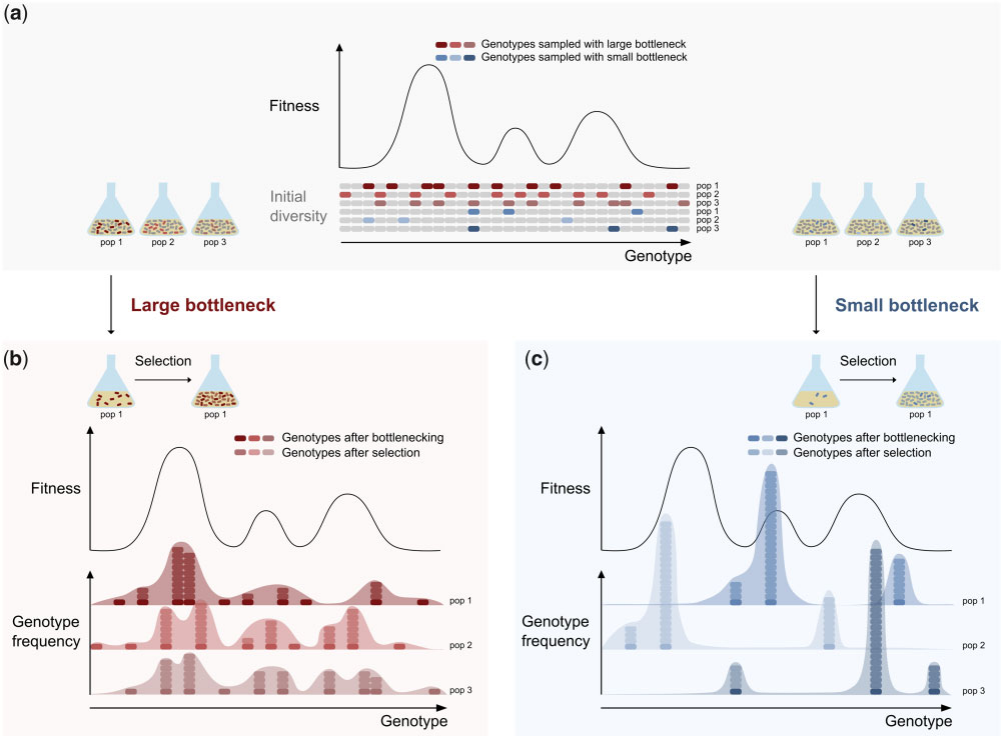
Bottlenecking reduces within-population genetic diversity and promotes between-population heterogeneity. Schematic overview of the effects of the bottleneck size on the genetic composition of parallel populations on a rugged fitness landscape (one-dimensional representation). (*a*) In evolution experiments and selection experiments with pooled libraries, initial genetic diversity is established through *de novo* mutations and pooling of mutants, respectively. These genotypes span the range of fitness levels that are accessible by mutation (gray cells). Before selection, all genotypes are present at approximately equal frequencies within a population. Hence, all genotypes have the same sampling probability during bottlenecking. When a large bottleneck is applied, more genotypes are sampled as compared with a small bottleneck (colored cells). (*b*) During selection, the genotypes sampled by bottlenecking increase in frequency according to their relative fitness (starting population is represented by the lower line of dark cells). In the case of a large bottleneck, a higher diversity of genotypes is sampled, resulting in populations that 1) show enrichment of high-fitness genotypes, 2) contain multiple genotypes with relatively high fitness and, consequently, relatively high frequency, and 3) show a genetic composition that is similar to parallel populations. (*c*) In the case of a small bottleneck, random sampling of a small number of genotypes results in populations that 1) show enrichment of potentially lower-fitness genotypes, 2) contain very few high-frequency genotypes and multiple genotypes with a low, but detectable frequency, and 3) show a genetic composition that is different from parallel populations.

The observed restricting effect of bottlenecking on the rate and extent of adaptation can be explained in two ways. First, small bottlenecks are associated with a strongly reduced mutational supply rate, which has previously been identified as an important determinant of the adaptation rate of small populations (De Visser et al. 1999). The distribution of fitness effects of beneficial mutations is generally assumed to be skewed, implying that only very few mutations generate considerable fitness gains (Fisher 1930; Orr 1998; Imhof and Schlötterer 2001; Rozen et al. 2002; Kassen and Bataillon 2006). A low mutational supply rate consequently involves restricted access to highly beneficial mutations. In our experiments, this limited mutational supply was apparent from the strongly reduced genetic diversity within populations exposed to small bottlenecks (fig. 6). Second, bottlenecking increases the extent of genetic drift, that is, the perturbation of genotype frequencies as a result of random events (Masel 2011). In contrast to selection, genetic drift does not act on fitness and hence results in the random fixation of deleterious mutations, as well as the loss of beneficial mutations (fig. 6) (Lynch et al. 1995; Lande 1998; Bergstrom et al. 1999; Burch and Chao 1999; Wahl and Gerrish 2001; Wahl et al. 2002).

We further observed that small bottlenecks do not only restrict the adaptive potential of populations, but also increase the between-population heterogeneity. This heterogeneity is reflected in the variability in persister levels as well as in the underlying genetic composition of populations, and can be explained by an increasing degree of randomness in the fixation of beneficial mutations (fig. 6) (Elena and Lenski 2003; De Visser and Rozen 2005; Rozen et al. 2008). Notably, this large heterogeneity among populations led to the identification of novel genes and ncRNAs that are potentially involved in persistence. These were found both by whole-genome sequencing of evolved populations and by sequencing the barcodes of genome-wide KO libraries that were subjected to two selection cycles. The limited overlap between both data sets suggests that most mutations found in evolved populations do not inflict a loss of function. Future research should reveal which of the observed mutations actually have a causal effect on persistence.

After 18 evolutionary cycles, adaptation to the antibiotic selection regime plateaued for most populations and showed a heterogeneous outcome. Although an in-depth investigation of gene interactions is required to obtain conclusive evidence, these results suggest that the persistence fitness landscape is presumably rugged, containing multiple peaks as a result of genetic epistasis (Weissman et al. 2009). As small-bottleneck populations scan the mutational space in a more stochastic manner, they might more readily end up at local, suboptimal fitness peaks that act as evolutionary traps (Poelwijk et al. 2007). Our data do not support hypotheses stating that small populations can attain higher fitness peaks than large populations on rugged fitness landscapes (Rozen et al. 2008; Salverda et al. 2017). However, this phenomenon might be strongly dependent on the selection regime and phenotype under study. Additionally, more parallel populations and/or longer evolution might be required in order to observe the rare populations that do reach these high fitness levels.

The current work yields important insights in the far-reaching effects of population bottlenecks. Importantly, the empirical evidence provided here involves the clinically relevant phenomenon of antibiotic persistence. Persistence is hypothesized to be one of the causes of antibiotic therapy failure, as persister cells survive antibiotic doses that are lethal to normal cells and thereby ensure the perpetuation of the bacterial population, resulting in recurrent and chronic infections (Brennan and Durack 1983; Mulcahy et al. 2010; Conlon et al. 2013; Claudi et al. 2014; Helaine et al. 2014; Kaiser et al. 2014). Moreover, recent work suggests that persistence could catalyze the emergence of resistance (Levin-Reisman et al. 2017; Sebastian et al. 2017; Bakkeren et al. 2019; Windels, Michiels, Fauvart, et al. 2019; Windels, Michiels, Van den Bergh, et al. 2019). For these reasons, understanding the evolutionary dynamics of persistence and the underlying mutational pathways is of vital importance. Here, we showed that population bottlenecks, which are frequently encountered by bacteria during the initiation and treatment of infections, govern the adaptation of populations to frequent antibiotic therapy. Our results do not only emphasize the implications of bottlenecking for persistence evolution, but also add to a growing set of genotypes underlying high persistence. Together, these observations confirm long-standing eco-evolutionary hypotheses and shed light on bacterial adaptation to antibiotic treatment.

## Materials and Methods

### Strains and Culture Conditions

Evolution experiments were initiated from an *E. coli* SX43 ancestor strain. This strain is identical to BW25993, except for the expression of a Tsr-Venus fusion from the *lacZ* locus (SX4), which results in yellow fluorescence at the cell poles (Yu et al. 2006). The kanamycin resistance cassette in the original strain was removed by Flp-mediated recombination, yielding the strain SX43 (Van den Bergh et al. 2016). Cultures were grown at 37 °C in Mueller-Hinton broth (MHB) with orbital shaking (200 rpm) or on lysogeny broth (LB) agar.

### Evolution Experiments

Distinct single colonies of SX43 were inoculated to start experimental evolution of parallel populations. Populations were grown in 96-well plates filled with 500 µl MHB until they reached a cell density of approximately 10^9^ colony-forming units per ml (CFU/ml), and treated daily for 5 h with 400 µg/ml amikacin, after transferring 200 µl to a fresh 96-well plate. After treatment, cultures were washed three times in 10 mM MgSO_4_ to remove the antibiotic. Washed cultures were diluted in MHB with different dilution factors (1:500–1:100–1:50–1:10; a dilution factor of 1:500 implies 1 unit of washed culture and 499 units of fresh medium), which correspond to the imposed bottleneck size. Applying more extreme bottlenecks resulted in 100% extinction of the populations. Forty parallel populations were initiated per bottleneck size. The cell density of the cultures was determined before and after each treatment by making 10-fold dilution series in MgSO_4_ followed by spot plating. Plates were incubated for 24 h before colonies were counted. The evolution experiment was continued for 18 cycles, corresponding to approximately 390 generations for 1:500 populations, 348 generations for 1:100 populations, 330 generations for 1:50 populations, and 288 generations for 1:10 populations. Before carrying out the actual evolution experiments, the prevalence of cross-contamination in this setup was monitored by evolving a small number of parallel populations initiated by multiple ancestral strains with different fluorescence properties. Cross-contamination was rarely observed.

### Determination of Minimum Inhibitory Concentrations

MICs were quantified based on the microdilution method (Wiegand et al. 2008). An overnight culture originating from a single colony was diluted in MHB to obtain a cell density of approximately 10^5^ CFU/ml. This inoculum was incubated for 24 h in a range of 2-fold antibiotic dilutions, after which growth was examined visually and quantified by measuring the optical density at 595 nm with a Synergy Mx Microplate Reader (BioTek). The MIC was defined as the lowest antibiotic concentration in which no growth was detected. Two clones were tested per population and for each clone, two technical replicates were included.

### Determination of Persister Fractions and Time-Kill Curves

Bacterial survival during antibiotic treatment was quantified by measuring the number of CFU per ml as a function of treatment duration. An overnight culture was diluted 1:100 in 500 µl MHB and again grown overnight for 16 h. This culture was then treated with 400 µg/ml amikacin. Cell densities were measured by washing samples in 10 mM MgSO_4_, making 10-fold dilution series in MgSO_4_, and plating on LB agar. For persistence assays, cell densities were determined before treatment and after 5 h of treatment. For time-kill curves, cell densities were measured before treatment and after 1, 2, 3, 5, and 8 h of treatment. Plates containing untreated cultures were incubated for 24 h, whereas plates containing treated cultures were incubated for 48 h before colonies were counted.

### Measurement of Growth Curves

Growth curves of evolved populations were measured by inoculating the populations in 500 µl MHB followed by 16 h of incubation (“monocultures”). These overnight cultures were then diluted 1:100 in 300 µl MHB and incubated for 19 h in a Bioscreen C incubator (37 °C, linear shaking) with automatic plate reading (600 nm) every 10 min (Bioscreen C, Oy growth curves). In order to measure competition coefficients, growth of evolved populations mixed with the ancestor was followed over time (“mixed cultures”). To this end, an overnight culture of every evolved population was mixed in a 50:50 ratio with an overnight culture of the ancestor strain, after correcting for differences in optical density. These mixtures were then diluted 1:100 in 300 µl MHB and incubated for 19 h in a Bioscreen C incubator (37 °C, linear shaking) with automatic plate reading (600 nm) every 10 min. Competition coefficients were derived from

(1) $${\mathrm{AUC}}_{\mathrm{mixed}}=\;{c\times \mathrm{AUC}}_{\mathrm{mono}}+\left(1-c\right)\times {\mathrm{AUC}}_{\mathrm{anc}},$$

where *c* equals the competition coefficient. AUC represents the area under the growth curve, with AUC_mixed_ the AUC of the mixed culture, AUC_mono_ the AUC of the monoculture and AUC_anc_ the AUC of the ancestral strain.

### Direct Competition Experiments

Evolved populations were competed against the ancestral strain to measure relative fitness levels. As the evolved populations expressed the Tsr-Venus fusion, competition experiments were performed using a nonfluorescent version of SX43 as the ancestor in order to distinguish it from evolved populations. An overnight culture of every evolved population was mixed in a 50:50 ratio with an overnight culture of the ancestor strain, after correcting for differences in optical density. The 50:50 ratio was verified by fluorescence microscopy. Next, the mixed cultures were diluted 1:100 and incubated for 16 h. The overnight cultures were treated for 5 h with 400 µg/ml amikacin, after which they were washed in 10 mM MgSO_4_, diluted 1:100 in fresh MHB and grown overnight. The frequency of each strain in the culture was determined before treatment and after the last overnight incubation, by counting the number of nonfluorescent and yellow fluorescent cells captured on images taken with a Nikon Ti-E inverted microscope with a 60× objective. At least five images were taken per culture per time point, containing between 600 and 2,700 cells in total.

### Whole-Genome Sequencing and Identification of Mutations

Genomic DNA was isolated from end populations using the DNeasy Blood and Tissue Kit (Qiagen). DNA purity and concentration were verified using NanoDrop and Qubit (Thermo Fisher). Libraries were prepared with the Nextera XT DNA Library Preparation Kit (Illumina). Whole-genome sequencing of evolved populations was either performed on an Illumina HiSeq 4000 at Nucleomics Core (Flemish Institute for Biotechnology) or on an Illumina NextSeq 500 at Genomics Core (University Hospital UZ Leuven) at 0.4–1 Gb per sample, corresponding to 87-217x coverage. Data analysis was performed on Linux. FastQC was used for quality control of the reads. Read trimming and filtering was performed with Trimmomatic 0.39 (adapters were removed, a sliding window was used to cut reads when the average base quality dropped below 20, reads shorter than 40 bases were discarded, and reads were only maintained when both reads of a pair passed through the filters). Reads were mapped to the *E. coli* MG1655 reference genome (NCBI accession number NC_000913.3) using Burrows-Wheeler Aligner (Li and Durbin 2009) with default parameters, and sorted and indexed with SAMtools 1.10 (Li et al. 2009). PCR duplicates were removed with Picard (http://broadinstitute.github.io/picard, last accessed 22/04/2021). Variants were called with VarScan v2.4.4 (Koboldt et al. 2012) and BCFtools 1.10 (Li 2011), and filtered with VarScan v2.4.4 and the SelectVariants tool of GATK (McKenna et al. 2010), respectively. Variant annotation was performed using SnpEff v4.3t (Cingolani et al. 2012).

### Selection Experiments with Onyx Libraries

#### Genome Engineering Libraries

Massively parallel genome engineered libraries were constructed using a developmental version of Inscripta’s Onyx Digital Genome Engineering platform. The Onyx platform precisely delivers single edits into genomes, resulting in barcoded, precisely engineered genome-scale cell libraries. The technology uses the MAD7 (cas12a class) CRISPR nuclease and guide RNA libraries similar to the CREATE method (Garst et al. 2017) to engineer single edits in cells. In summary, populations of editing cassettes were constructed where each cassette encodes a specific CRISPR guide RNA, an associated donor DNA sequence which imparts the desired edit at the genomic location of interest, and a unique barcode sequence. Editing cassettes were cloned in bulk into a high copy plasmid backbone, enabling genome editing coupled to plasmid-based barcode tracking of edit designs. Editing cassette-encoding plasmid populations were then transformed into MAD7-expressing cells, and genomic editing was performed using developmental reagents and protocols. For this study, a library containing 87 different ncRNA KO mutants was constructed in *E. coli* strain SX43 by deleting the first 30 nucleotides of each nonessential ncRNA. The fraction of edited cells in the library was determined to be 36% by whole-genome shotgun sequencing (Illumina NextSeq 550) of 96 randomly sampled members of the population. Additionally, a genome-wide KO library on 4,128 annotated loci was constructed by introducing an in-frame premature stop codon at the 15th codon of each nonessential gene in the SX43 background. The fraction of edited cells in the library was determined to be 17% by whole-genome shotgun sequencing of 96 randomly sampled members of the population.

#### Selection Protocol

The Onyx libraries were subjected to the same selection regime that was used during the evolution experiments. Overnight cultures were treated for 5 h with a high dose of amikacin (400 µg/ml), after which they were washed and diluted in fresh MHB. Dilution factors of 1:10, 1:50, 1:100, 1:500, 1:1,000, and 1:5,000 were applied, corresponding to the imposed bottleneck. Per bottleneck, eight parallel populations were initiated from each of the two libraries. The cycle of treatment, dilution, and overnight growth was repeated twice.

#### Barcode Amplicon Sequencing

The representation of all designs in the libraries was tracked by deep sequencing of the barcode region of the plasmid population. Library samples were collected at the designated time points and DNA was extracted using the Wizard SV Genomic DNA Purification System (Promega). For barcode amplicon PCR, extracted DNA was diluted to 2 ng/μl with EB buffer (Qiagen), and 5 μl was used as template in a 50-μl PCR reaction consisting of 25 μl 2X Q5 Hot Start High-Fidelity DNA Polymerase Master Mix (NEB), 15 μl nuclease-free water, and 5 μl primer mix (1 μM final each). PCR was initiated with 2 min of incubation at 98 °C, followed by 20 cycles at 98 °C for 10 s and 72 °C for 2 min and 30 s, a 5 min hold at 72 °C, and finishing with a hold at 4 °C. PCR products were then sequenced on an Illumina NextSeq 550 and an Illumina Miseq at Inscripta.

#### Control Experiment

The above-described selection protocol does not only favor strains with increased persistence, but is also expected to select for strains with affected growth characteristics in antibiotic-free medium. In order to distinguish both types of mutants, a control experiment was performed in which the Onyx libraries were grown overnight. Eight parallel populations were initiated from each of the two libraries. The frequency of mutants in each population was determined before and after selection by PCR amplification of the barcode region of the editing plasmid population and deep sequencing on an Illumina NextSeq 550 and an Illumina Miseq at Inscripta.

### Data Analysis

#### Statistical Analysis

Correlations between the bottleneck size and any quantitative property were analyzed by performing Spearman rank correlation tests. Statistical comparison of MIC values was done with unpaired two-sided *t-*tests with Welch’s correction in the case of unequal variances (checked with an *F*-test). The *P* values were corrected for multiple comparisons using the Benjamini–Hochberg method (Benjamini and Hochberg 1995). The fitness of evolved populations was compared with the fitness of the ancestor with a two-sided one-sample *t*-test on the relative fitness (H_0_: *µ* = 1). The AUC of evolved populations grown in monocultures was compared with the AUC of the ancestral strain with a two-sided *t*-test with Welch’s correction because of unequal variances (checked with an *F*-test). The *P* values were corrected for multiple comparisons using the Benjamini–Hochberg method. The correlation between the composition of parallel Onyx populations was determined by calculating for each pair of populations the Pearson correlation coefficient based on the raw read counts of all mutants. The number of lost mutants per population was calculated as the number of mutants with a read count lower than three after selection. Distributions of mutant frequencies in evolved populations and Onyx populations were smoothed using the R package *ggridges* (https://cran.r-project.org/web/packages/ggridges/index.html, last accessed 22/04/2021).

#### Fittings on Evolutionary Trajectories

To describe evolutionary dynamics, a sigmoidal model with equation

(2) $$p=\;\frac{1}{1+\;e^{-S(t-\lambda )}}$$

was fitted onto the time course of the log_10_-transformed persister fractions using the R package *nlstools* (https://cran.r-project.org/web/packages/nlstools/index.html, last accessed 22/04/2021). In equation (2), *p* equals the log_10_-transformed persister fraction, *S* reflects the steepness of the sigmoidal increase (i.e., rate of adaptation), and *λ* represents the lag time before adaptation occurs (i.e., lag of adaption). These fittings were either performed on trajectories of individual populations or on all populations evolved with the same bottleneck.

#### Simulation of Evolutionary Trajectories

Evolutionary trajectories describing the spread of a single mutant in an ancestral population (fig. 2*b*) were simulated based on the following discrete-time recurrence equation (Otto and Day 2007):

(3) $$p\left(T+1\right)=\;\frac{W_{A}\;\times \;p(T)}{W_{A}\;\times \;p\left(T\right)+q(T)},$$

where *W_A_* represents the fitness of the mutant relative to the ancestral strain and *p*(*T*) and *q*(*T*) are the frequencies of the mutant and ancestral strain respectively, after *T* selection rounds. In order to account for bottlenecking, a correction factor $D(\ln D{)}^{2}$ was applied, in which *D* represents the dilution ratio (Wahl et al. 2002). An initial mutant frequency of 10^−8^ was assumed, corresponding to one mutant cell in a population of 10^8^ cells.

#### Read Count Analysis of Onyx Libraries

The R package *DESeq2* was used to compare read counts in the Onyx libraries before and after selection and identify significant enrichments or depletions (Love et al. 2014). For the ncRNA KO library, raw read counts were normalized using the read counts of three different inert designs. For the gene KO library, normalization required more control genes to compensate for the higher between-replicate variability. In this case, 15 silent genes from the *blg* operon, the *asc* operon, and the *chb* operon were used to normalize the read counts. Mutants with potentially affected antibiotic tolerance were distinguished from mutants with affected growth, by filtering out the significant hits from the control experiment.

## Supplementary Material

Supplementary data are available at *Molecular Biology and Evolution* online.

## Supplementary Material

msab107_Supplementary_Data
