# Supplementary material for: Population Bottlenecks Strongly Affect the Evolutionary Dynamics of Antibiotic Persistence
Source: Mol Biol Evol. 2021 Apr 19;38(8):3345–57. doi: 10.1093/molbev/msab107 (PMC8321523; doi:10.1093/molbev/msab107)
Supplement: msab107_Supplementary_Data [file msab107_supplementary_data.pdf]

# Supplementary figures

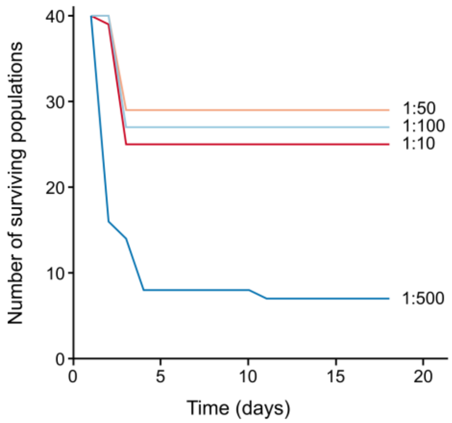


**Figure S1. Small bottlenecks result in high extinction rates during experimental evolution.** Number of populations surviving the evolution experiment, which was initiated with 40 parallel populations per condition. Populations subjected to the strongest dilution during serial transfer show the highest extinction rate. Most populations went extinct during the first three rounds of selection, while populations that survived those first cycles were mostly sustained.

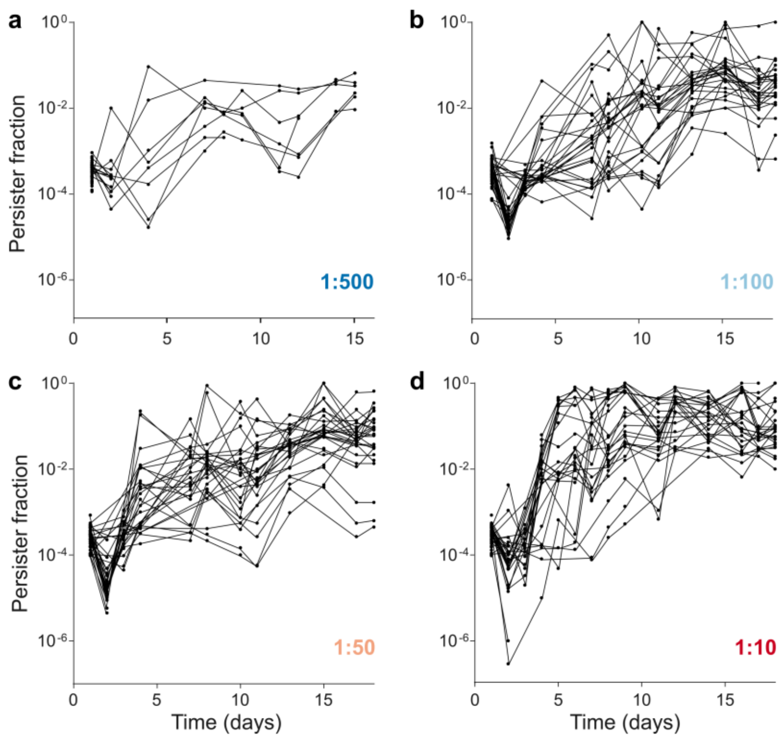


**Figure S2. Persistence rapidly evolves under intermittent antibiotic exposure.** *E. coli* populations were evolved under daily, high-dose amikacin treatment intermitted with growth periods to reach stationary phase, with varying population bottleneck sizes. Bottlenecks were imposed through dilution during serial transfer, with 1:500 (a), 1:100 (b), 1:50 (c), and 1:10 (d) dilution factors. In all conditions, the fraction of cells surviving the antibiotic treatment rapidly increased over time. Data points represent a single measurement per population.

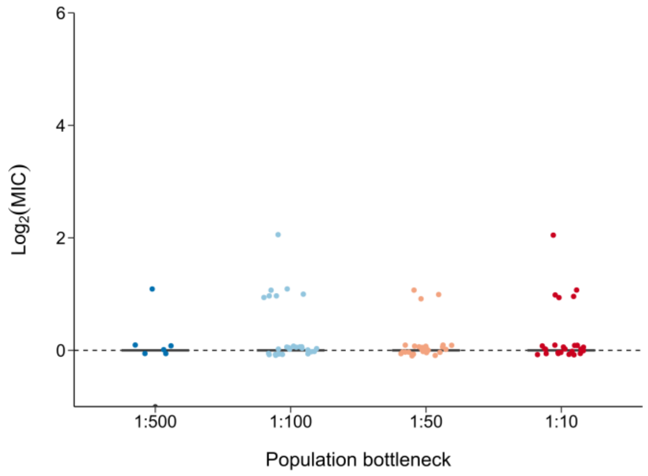


**Figure S3. The minimum inhibitory concentration (MIC) of evolved populations is not significantly increased.** The MIC was measured for two clones of each evolved population, and the average MIC per population is shown. While two populations show slightly increased MIC values, the MIC of evolved populations is not significantly different from the MIC of the ancestral strain (dashed line; Student's *t*-tests with correction for multiple testing: 1:500: p=0.99, 1:100: p=0.99, 1:50: p=0.85, 1:10: p=0.35).

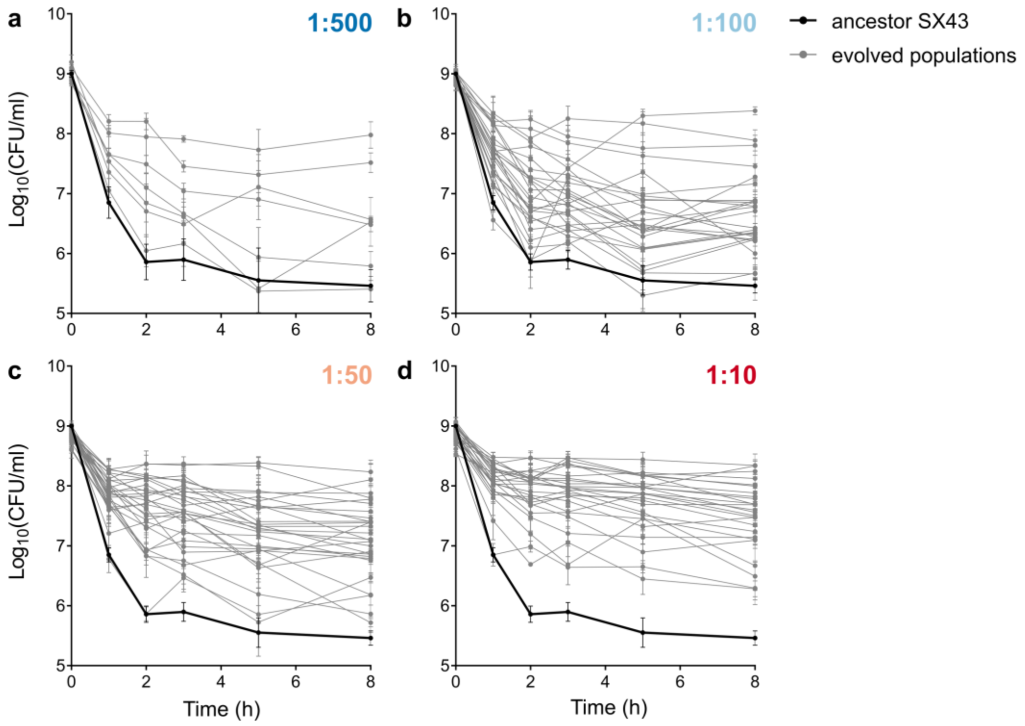


**Figure S4. Time-kill curves of evolved populations are biphasic.** The time-kill curves measured for 3 clones of each evolved population show a biphasic pattern reflecting persistence. The curves of evolved populations (grey) are characterized by a high persister plateau as compared to the ancestor (black).

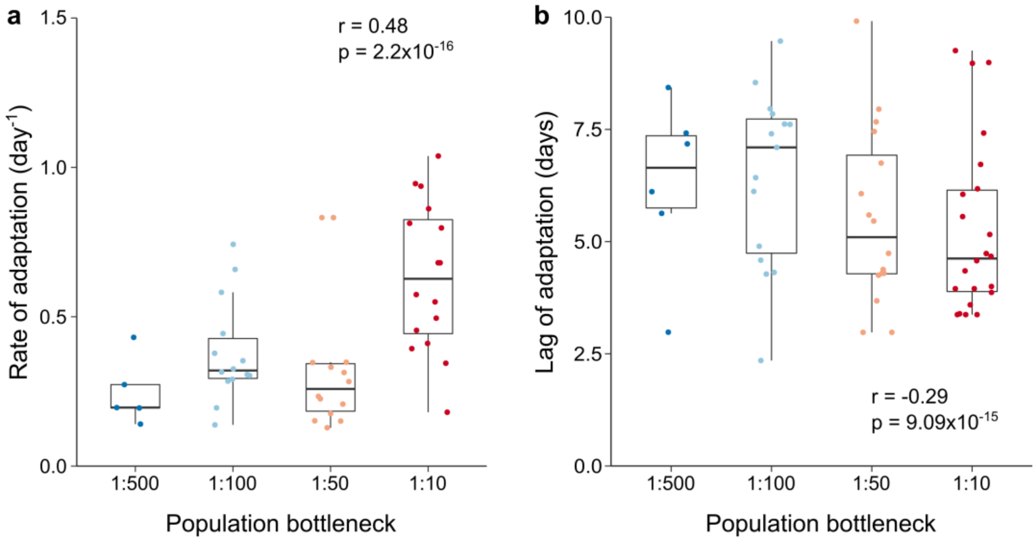


**Figure S5. Parameters of evolutionary dynamics are correlated with the bottleneck size.** A sigmoidal model was fitted onto the evolutionary trajectory of each population (see Methods) and the parameter estimates reflecting the rate (a) and lag (b) of adaptation are represented. The bottleneck size is positively correlated with the rate of adaptation (Spearman rank correlation: $r = 0.48$; $p = 2.2x10^{-16}$) and negatively correlated with the lag of adaptation (Spearman rank correlation: $r = -0.29$; $p = 9.09x10^{-15}$).

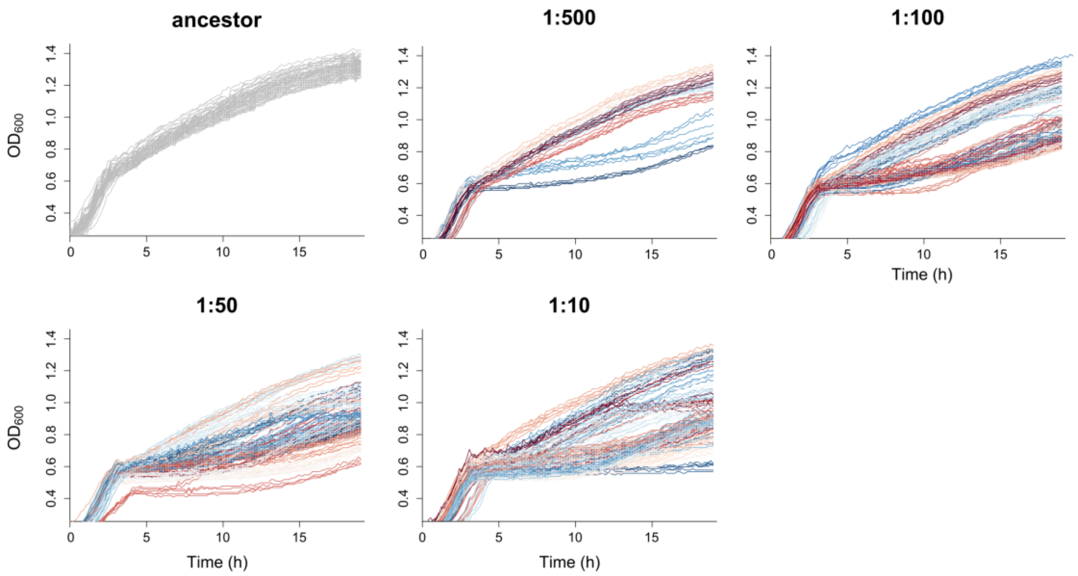


**Figure S6. Growth curves of evolved populations show impaired growth compared to the ancestor.** Growth of evolved populations was followed over time by measuring the optical density at 600 nm ($OD_{600}$) in antibiotic-free medium. Different colors represent different evolved populations. 6 biological replicates were measured per population and are represented by the same color.

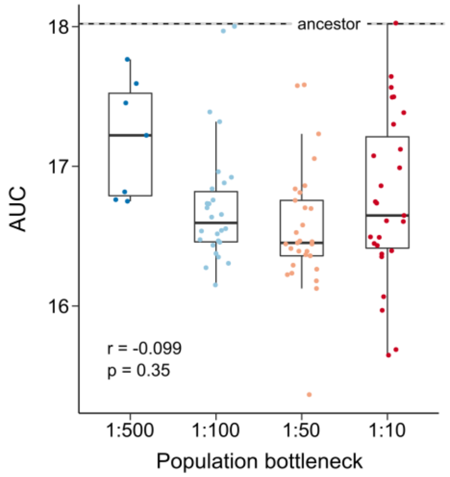


**Figure S7. The AUC of growth curves of evolved populations mixed with the ancestor negatively correlates with the bottleneck size.** The AUC calculated from growth curves of mixed cultures (50:50) decreases with increasing bottleneck size, although this correlation lacks statistical support due to the presence of the ancestral strain in all cultures (Spearman rank correlation: r = -0.099; p = 0.35) (dashed line: mean of ancestral monoculture; grey shading: 95 % CI of ancestral monoculture).

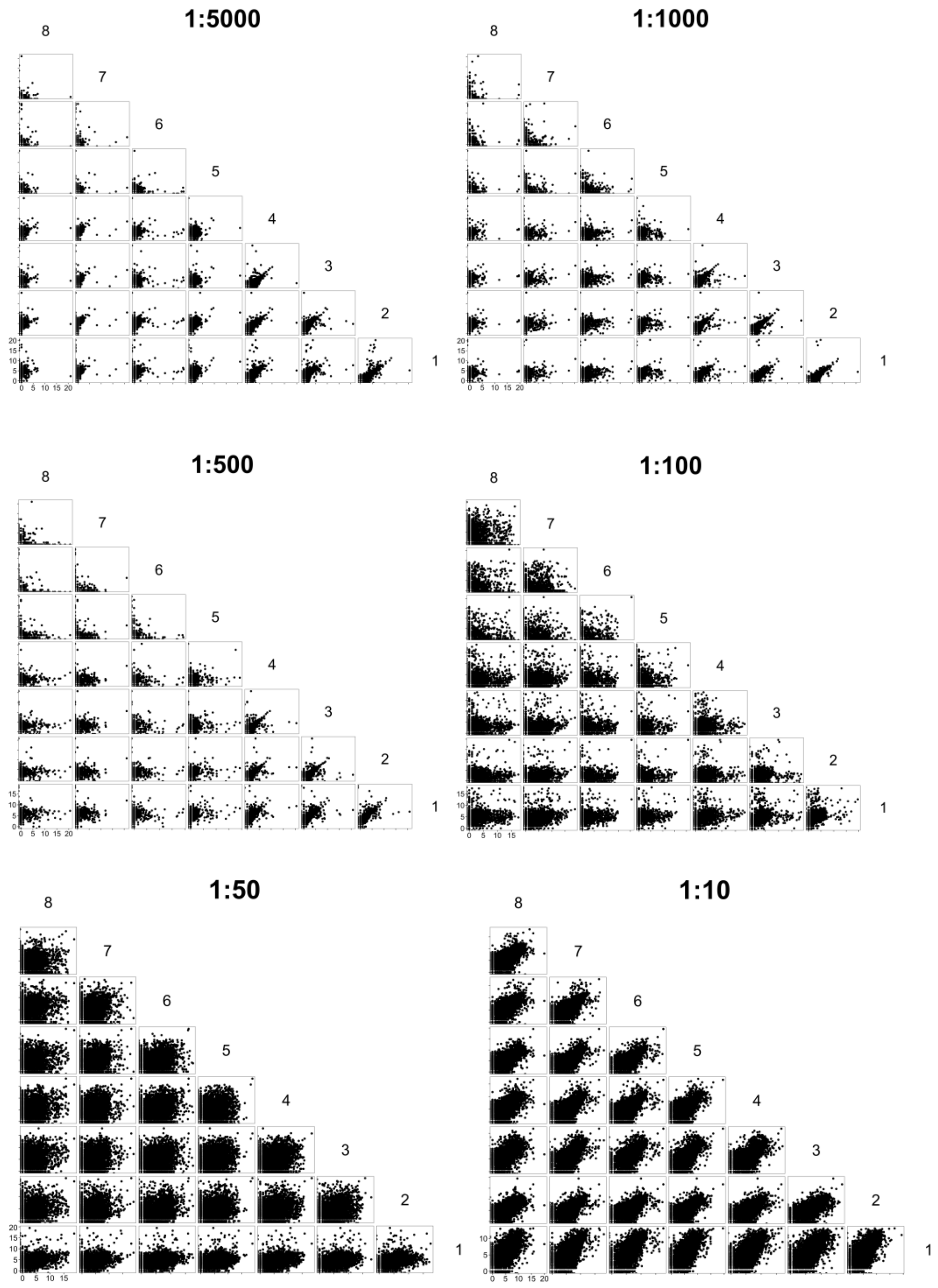


**Figure S8. Bottlenecking promotes between-population heterogeneity in gene KO libraries.** Pairwise scatterplots of $Log_2$-transformed raw read counts of parallel populations, obtained by subjecting gene KO libraries to two rounds of antibiotic selection. Correlations increase with increasing bottleneck size.

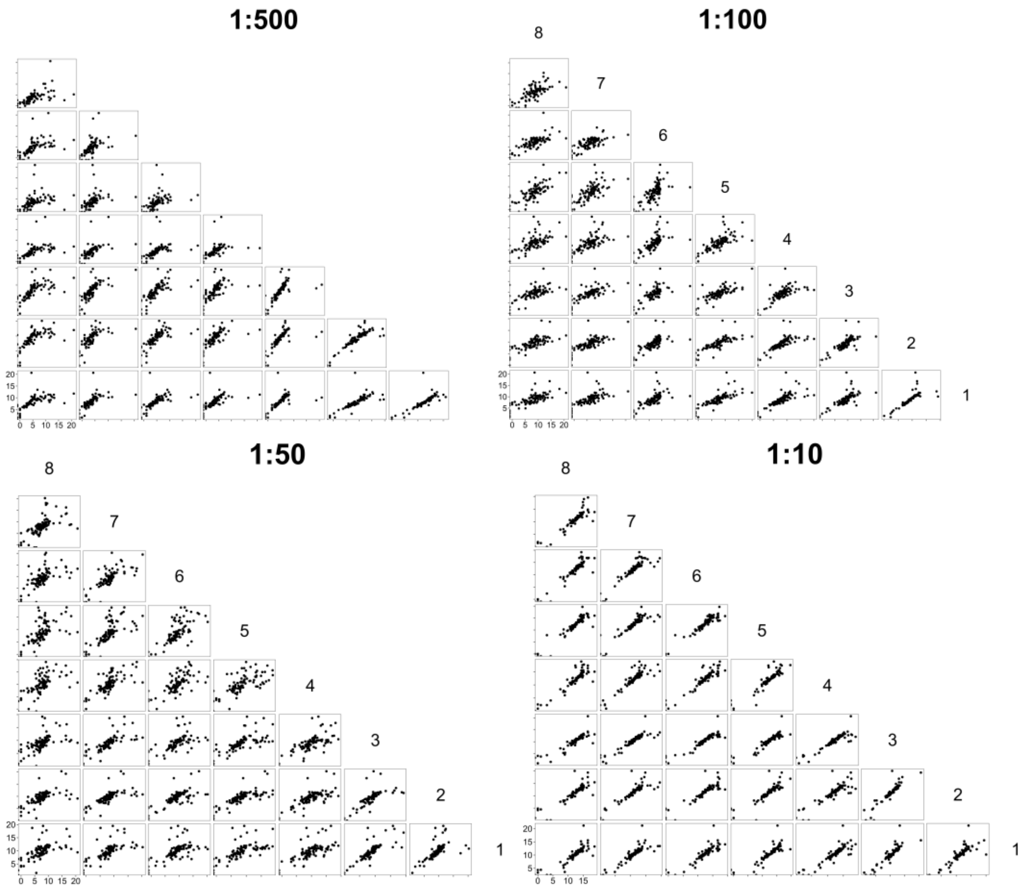


**Figure S9. Bottlenecking promotes between-population heterogeneity in ncRNA KO libraries.** Pairwise scatterplots of $Log_2$-transformed raw read counts of parallel populations, obtained by subjecting ncRNA KO libraries to two rounds of antibiotic selection. Correlations increase with increasing bottleneck size. Selection regimes with bottlenecks corresponding to 1:5000 and 1:1000 dilution led to population extinction.

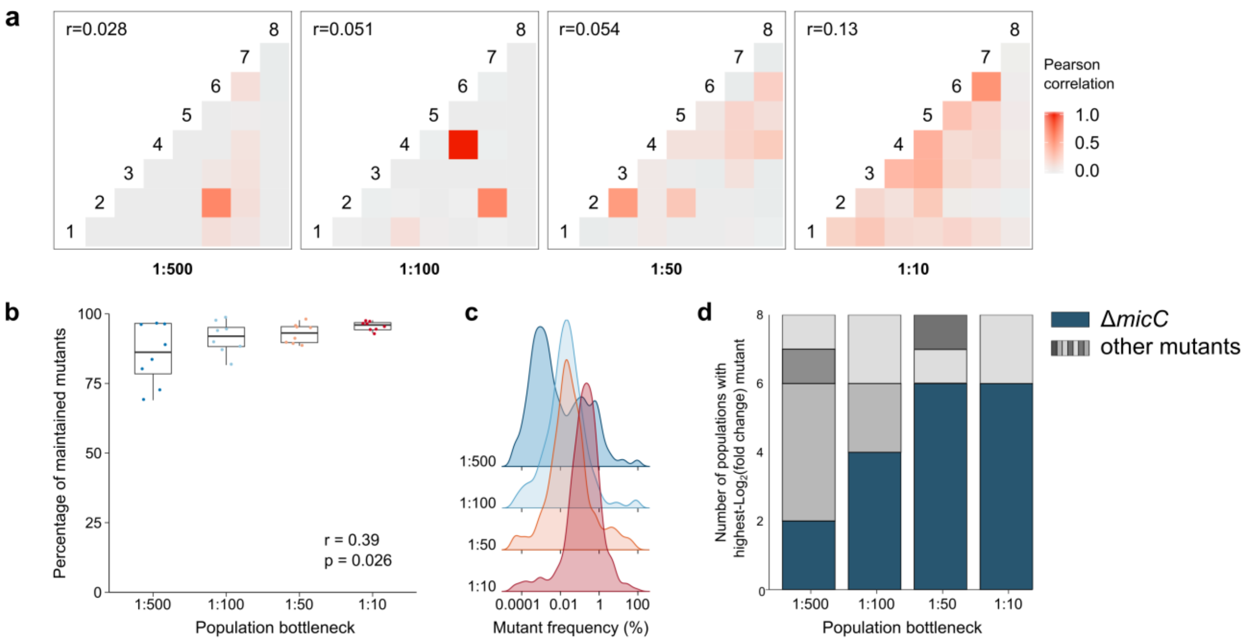


**Figure S10. Bottlenecking affects the composition of genome-wide ncRNA KO libraries after antibiotic selection and promotes between-population heterogeneity.** (a) Correlation plots of the genetic composition of 8 parallel populations, obtained by subjecting ncRNA KO libraries to two rounds of antibiotic selection. The composition of the populations after selection was determined by calculating the frequency of each mutant based on raw sequencing read counts. Small population bottlenecks result in weaker correlations between parallel populations (r = Pearson correlation coefficient averaged over all comparisons). No negative correlations were observed. Selection regimes with bottlenecks corresponding to 1:5000 and 1:1000 dilution led to population extinction. (b) The genetic diversity, represented as the percentage of mutants maintained in a population after selection, is positively correlated with the bottleneck size (Spearman rank correlation: r = 0.39; p = 0.026). The complexity of the ncRNA KO library is low compared to the gene KO library, resulting in fewer mutants lost by bottlenecking. (c) Distributions of mutant frequencies within populations. The peak height represents the number of mutants with a certain frequency within a population, normalized over the total number of populations. Small-bottleneck populations mainly contain low-frequency mutants, while the distribution shifts to higher frequencies as the bottleneck increases. (d) Number of populations in which a certain mutant shows the highest absolute

$Log_2$(fold change) after selection, based on raw read counts. Different colors represent different mutants. Parallel populations exposed to large bottlenecks often contain similar mutants, while small-bottleneck populations show a larger between-population heterogeneity (Spearman rank correlation: $r = 0.95$; $p = 0.05$).

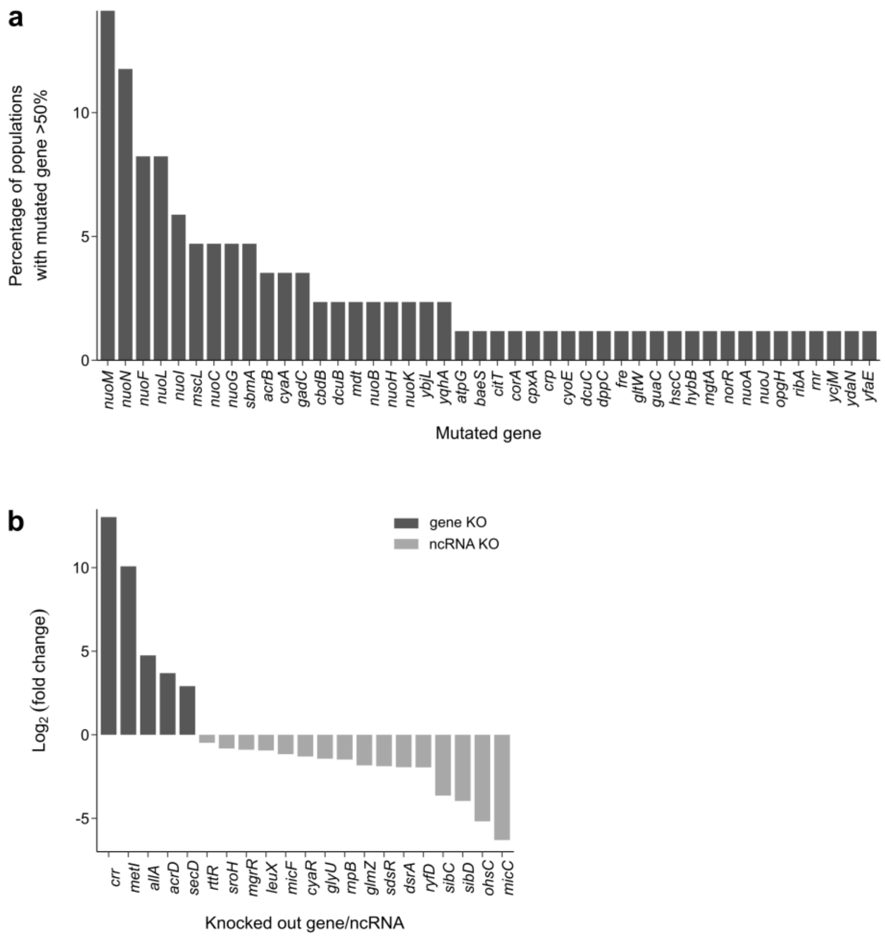


**Figure S11. Experimental evolution and selection experiments with barcoded knockout libraries reveal genes that are potentially involved in persistence.** (a) Overview of mutations that attained a frequency of 50 % or higher in experimentally evolved populations. The height of the bars represents the percentage of populations in which the corresponding gene was mutated at high frequency. (b) Overview of mutations that showed a significant $Log_2$(fold change) after subjecting gene KO or ncRNA KO libraries to antibiotic selection. Analysis was based on DESeq2 normalized read counts.

# Supplementary tables

**Table S1**. Variants with a population frequency of 50 % or higher, as identified by whole-genome sequencing of evolved populations

| Bottleneck | Gene | Location | Mutation | Amino acid change |
|---|---|---|---|---|
| 1:500 | *nuoA* | 2404819 | 254T>G | Leu85Arg |
| 1:500 | *nuoM* | 2392613 | 426_428delCTG | Phe142_Trp143delinsLeu |
| 1:500 | *nuoM* | 2392614 | 425_427delTCT | Phe142del |
| 1:500 | *opgH* | 1111464 | 602T>G | Leu201Arg7 |
| 1:100 | *acrB* | 481493 | 2911C>T | Arg971Cys |
| 1:100 | *acrB* | 481837 | 2567G>A | Gly856Asp |
| 1:100 | *cbdB* | 1039576 | 281T>G | Leu94Arg |
| 1:100 | *cyaA* | 3992052 | 900T>G | Asp300Glu |
| 1:100 | *dcuB* | 4347813 | 932T>A | Val311Glu |
| 1:100 | *gadC* | 1569390 | 1100T>C | Phe367Ser |
| 1:100 | *guaC* | 114107 | 664T>G | Cys222Gly |
| 1:100 | *hscC* | 682570 | 824T>G | Leu275Arg |
| 1:100 | *nuoC* | 2402588 | 1257delC | Thr420fs |
| 1:100 | *nuoC* | 2402633 | 1213C>T | Gln405* |
| 1:100 | *nuoC* | 2403813 | 33G>A | Trp11* |
| 1:100 | *nuoF* | 2401336 | 219dupG | Ser74fs |
| 1:100 | *nuoF* | 2401513 | 43T>G | Trp15Gly |
| 1:100 | *nuoH* | 2396788 | 655C>T | Gln219* |
| 1:100 | *nuoI* | 2396356 | 88_94delACGCGAA | Thr30fs |
| 1:100 | *nuoI* | 2396449 | 2T>G | Met1? |
| 1:100 | *nuoL* | 2394292 | 755T>G | Leu252Arg |
| 1:100 | *nuoL* | 2394362 | 685A>C | Lys229Gln |
| 1:100 | *nuoM* | 2392017 | 1025T>A | Val342Glu |
| 1:100 | *nuoM* | 2392323 | 719T>G | Leu240Arg |
| 1:100 | *nuoM* | 2392614 | 425_427delTCT | Phe142del |
| 1:100 | *nuoN* | 2390118 | 1388T>A | Leu463* |
| 1:100 | *nuoN* | 2390604 | 902C>A | Ser301* |
| 1:100 | *ribA* | 1338580 | 581T>G | Leu194Arg |
| 1:100 | *sbmA* | 397099 | 461T>G | Leu154Arg |
| 1:50 | *cbdB* | 1039576 | 281T>G | Leu94Arg |
| 1:50 | *citT* | 645712 | 869T>G | Leu290Arg |
| 1:50 | *corA* | 4001574 | 149T>G | Leu50Arg |
| 1:50 | *crp* | 3486750 | 631T>C | Ter211Glnext*? |
| 1:50 | *cyaA* | 3993163 | 2011C>T | Gln671* |
| 1:50 | *dcuB* | 4347813 | 932T>A | Val311Glu |
| 1:50 | *dcuC* | 655019 | 950T>G | Leu317Arg |
| 1:50 | *dppC* | 3704307 | 455T>C | Val152Ala |
| 1:50 | *fre* | 4026542 | 16T>C | Cys6Arg |

| | | | | |
|---|---|---|---|---|
| 1:50 | *gadC* | 1569376 | 1111_1113delCTG | Leu371del |
| 1:50 | *gltW* | 2729426 | 19C>T | Pro7Ser |
| 1:50 | *hybB* | 3143833 | 332G>A | Gly111Asp |
| 1:50 | *mscL* | 3438087 | 64G>A | Gly22Ser |
| 1:50 | *nuoB* | 2404314 | 299delG | Gly100fs |
| 1:50 | *nuoC* | 2403152 | 694C>T | Gln232* |
| 1:50 | *nuoF* | 2400503 | 1053C>A | Cys351* |
| 1:50 | *nuoF* | 2400999 | 557T>G | Leu186* |
| 1:50 | *nuoG* | 2398291 | 1874delG | Gly625fs |
| 1:50 | *nuoG* | 2398445 | 1719_1720insC | His574fs |
| 1:50 | *nuoI* | 2396272 | 179G>T | Cys60Phe |
| 1:50 | *nuoI* | 2396356 | 79_85delAAACGCG | Thr30fs |
| 1:50 | *nuoK* | 2395147 | 199A>C | Ser67Arg |
| 1:50 | *nuoK* | 2395209 | 121_132delGCCTCCGCGCTG | Ala41_Leu44del |
| 1:50 | *nuoL* | 2394292 | 755T>G | Leu252Arg |
| 1:50 | *nuoL* | 2394362 | 685A>C | Lys229Gln |
| 1:50 | *nuoL* | 2394599 | 448T>C | Ser150Pro |
| 1:50 | *nuoM* | 2392323 | 719T>G | Leu240Arg |
| 1:50 | *nuoM* | 2392567 | 470delG | His159fs |
| 1:50 | *nuoM* | 2392614 | 425_427delTCT | Phe142del |
| 1:50 | *nuoN* | 2390085 | 1421T>G | Leu474Arg |
| 1:50 | *nuoN* | 2390478 | 1013_1021delTCGGCGCGT | Gly339_Phe341del |
| 1:50 | *nuoN* | 2390577 | 929T>G | Leu310Arg |
| 1:50 | *norR* | 2832052 | 238G>A | Ala80Thr |
| 1:50 | *rnr* | 4407090 | 437T>A | Val146Glu |
| 1:50 | *sbmA* | 397399 | 761G>A | Gly254Glu |
| 1:50 | *ycjM* | 1370267 | 52A>G | Thr18Ala |
| 1:50 | *ydaN* | 1408870 | 821C>A | Ala274Glu |
| 1:50 | *yfaE* | 2348767 | 254G>A | Ter85Ter |
| 1:50 | *yqhA* | 3149124 | 348T>G | Ile116Met |
| 1:10 | *acrB* | 481492 | 2912G>A | Arg971His |
| 1:10 | *atpG* | 3917541 | 725C>A | Ala242Asp |
| 1:10 | *atpG* | 3917542 | 724G>C | Ala242Pro |
| 1:10 | *baeS* | 2163770 | 895T>C | Ser299Pro |
| 1:10 | *cpxA* | 4104815 | 161A>C | Glu54Ala |
| 1:10 | *cyoE* | 447654 | 52A>G | Asn18Asp |
| 1:10 | *gadC* | 1569390 | 1100T>C | Phe367Ser |
| 1:10 | *mdtC* | 2159199 | 814G>A | Ala272Thr |
| 1:10 | *mdtF* | 3663318 | 2905C>T | Arg969Cys |
| 1:10 | *mgtA* | 4470032 | 2408A>T | Gln803Leu |
| 1:10 | *mscL* | 3438087 | 64G>A | Gly22Ser |
| 1:10 | *mscL* | 3438117 | 94A>G | Ile32Val |
| 1:10 | *nuoB* | 2404407 | 206delT | Val69fs |
| 1:10 | *nuoF* | 2400873 | 683T>G | Ile228Ser |
| 1:10 | *nuoF* | 2401266 | 290G>T | Gly97Val |
| 1:10 | *nuoG* | 2399028 | 1138G>T | Glu380* |

| | | | | |
|---|---|---|---|---|
| 1:10 | *nuoG* | 2399975 | 190dupG | Val64fs |
| 1:10 | *nuoH* | 2397015 | 428T>A | Leu143* |
| 1:10 | *nuoI* | 2396132 | 319G>T | Glu107* |
| 1:10 | *nuoJ* | 2395842 | 47_54dupCCTTGCGA | Val19fs |
| 1:10 | *nuoL* | 2394021 | 1026A>C | Lys342Asn |
| 1:10 | *nuoL* | 2394166 | 881C>A | Ala294Asp |
| 1:10 | *nuoM* | 2392017 | 1025T>A | Val342Glu |
| 1:10 | *nuoM* | 2392323 | 719T>G | Leu240Arg |
| 1:10 | *nuoM* | 2392614 | 425_427delTCT | Phe142del |
| 1:10 | *nuoN* | 2390476 | 1020_1028delGTTCGGCGT | Phe341_Val343del |
| 1:10 | *nuoN* | 2390478 | 1028T>C | Val343Ala |
| 1:10 | *nuoN* | 2390517 | 989T>G | Leu330Arg |
| 1:10 | *nuoN* | 2390577 | 929T>G | Leu310Arg |
| 1:10 | *nuoN* | 2391503 | 3G>T | Met1? |
| 1:10 | *sbmA* | 396934 | 296G>A | Trp99* |
| 1:10 | *sbmA* | 397597 | 959A>G | Asn320Ser |
| 1:10 | *ybjL* | 889419 | 401T>A | Val134Asp |
| 1:10 | *yqhA* | 3149135 | 337A>C | Ile113Leu |

**Table S2.** ncRNA KO mutants showing a significant $Log_2$(fold change) after selection, based on DESeq2 normalized read counts (lfcSE: standard error of $Log_2$(fold change); $p_{adj}$: adjusted p-value)

| **Bottleneck** | **Gene** | **$Log_2$(fold change)** | **lfcSE** | **$p_{adj}$** |
|---|---|---|---|---|
| 1:500 | *ohsC* | -6.28 | 1.66 | $3.47 \times 10^{-3}$ |
| 1:100 | *micC* | -6.30 | 1.86 | $1.62 \times 10^{-2}$ |
| 1:100 | *sibD* | -4.35 | 1.34 | $2.12 \times 10^{-2}$ |
| 1:10 | *ohsC* | -4.08 | 0.61 | $3.34 \times 10^{-10}$ |
| 1:10 | *sibC* | -3.64 | 0.36 | $9.55 \times 10^{-22}$ |
| 1:10 | *sibD* | -3.57 | 0.49 | $6.03 \times 10^{-12}$ |
| 1:10 | *ryfD* | -1.95 | 0.43 | $4.56 \times 10^{-5}$ |
| 1:10 | *dsrA* | -1.94 | 0.31 | $3.64 \times 10^{-9}$ |
| 1:10 | *sdsR* | -1.88 | 0.31 | $1.04 \times 10^{-8}$ |
| 1:10 | *glmZ* | -1.83 | 0.27 | $1.92 \times 10^{-10}$ |
| 1:10 | *rnpB* | -1.48 | 0.37 | $3.78 \times 10^{-4}$ |
| 1:10 | *glyU* | -1.43 | 0.34 | $2.08 \times 10^{-4}$ |
| 1:10 | *cyaR* | -1.30 | 0.24 | $7.56 \times 10^{-7}$ |
| 1:10 | *micF* | -1.16 | 0.29 | $3.78 \times 10^{-4}$ |
| 1:10 | *mgrR* | -1.03 | 0.21 | $7.63 \times 10^{-6}$ |
| 1:10 | *leuX* | -0.94 | 0.31 | $1.24 \times 10^{-2}$ |
| 1:10 | *sroH* | -0.82 | 0.28 | $1.29 \times 10^{-2}$ |
| 1:10 | *mgrR* | -0.76 | 0.33 | $7.10 \times 10^{-3}$ |
| 1:10 | *rttR* | -0.48 | 0.16 | $1.24 \times 10^{-2}$ |

**Table S3**. Gene KO mutants showing a significant $Log_2$(fold change) after selection, based on DESeq2 normalized read counts (lfcSE: standard error of $Log_2$(fold change); $p_{adj}$: adjusted p-value)

| **Bottleneck** | **Gene** | **$Log_2$(fold change)** | **lfcSE** | **$p_{adj}$** |
|---|---|---|---|---|
| 1:5000 | *crr* | 11.44 | 1.59 | $2.81 \times 10^{-9}$ |
| 1:5000 | *acrD* | 3.69 | 0.75 | $1.90 \times 10^{-3}$ |
| 1:5000 | *allA* | 4.75 | 0.98 | $1.90 \times 10^{-3}$ |
| 1:5000 | *secD* | 2.91 | 0.67 | $1.00 \times 10^{-2}$ |
| 1:1000 | *crr* | 11.08 | 1.96 | $6.67 \times 10^{-5}$ |
| 1:100 | *crr* | 15.74 | 3.55 | $3.80 \times 10^{-2}$ |
| 1:50 | *crr* | 16.30 | 2.36 | $1.85 \times 10^{-8}$ |
| 1:50 | *metI* | 10.08 | 2.38 | $4.80 \times 10^{-2}$ |
| 1:10 | *crr* | 10.57 | 1.19 | $2.49 \times 10^{-15}$ |
